# Physical performance profiling in Moroccan women’s football: a functional assessment from the Rabat-Salé-Kénitra regional league

**DOI:** 10.1186/s13102-025-01453-3

**Published:** 2025-12-03

**Authors:** Mohammed Benhida, Lotfi Zeghari, Khalid El Mouahid, Said El Morchidy, Youssef El Madhi, Nourddine Enneya, Fatima-Zahra Guerss

**Affiliations:** 1 Research Laboratory in Computer Science, Faculty of Sciences, Ibn Tofail University, Kénitra, Morocco; 2 Sport Science Institute, Sidi Mohamed Ben Abdellah University, Fez, Morocco; 3 Laboratory of Sport Sciences and Performance Optimization, Royal Institute for Training Cadres (IRFC), Salé, Morocco; 4 Laboratory for Education, Environment and Health, Regional Center for Education and Training (CRMEF), Rabat, Morocco

**Keywords:** Endurance, Sprint, Optojump, Performance testing, Athlete monitoring

## Abstract

**Background:**

This study aimed to establish normative physical performance profiles for Moroccan female football players across three competition levels and four age categories. Despite the recent growth of women’s football in Morocco, evidence-based data on physical capacities remain scarce. Developing standardized performance benchmarks is essential for guiding talent identification and individualized training strategies.

**Methods:**

A total of 109 female footballers (aged 14–32 years), from five clubs competing across three levels of the Moroccan national championship, underwent a standardized test battery including sprint tests (10, 20, 30 m), coordination sprint (20 m slalom), countermovement jump with and without arm swing (CMJ and CMJmax), squat jump (SJ), drop jump (DJ), a 15 s repeated jump test, horizontal jump, 30-second abdominal test, medicine-ball throw, Vameval test for maximal aerobic speed (MAS), and Ruffier–Dickson recovery test. Players were grouped by competitive level, age category, and playing position. Reliability analysis in a sub-sample (*n* = 15) confirmed high reproducibility (ICC = 0.87–0.95; CV = 3.8–5.8%). Group comparisons were conducted using ANOVA/ANCOVA, with age as a covariate and partial eta-squared (η²p) as effect size.

**Results:**

Significant age effects were found for CMJ-BL (*p* = 0.021, η²*p* = 0.105) and MAS (*p* = 0.001, η²*p* = 0.175), with senior players outperforming younger categories, while 10 m sprint times did not differ (*p* = 0.147). Across competition levels, second-division players showed superior vertical and horizontal jumps (*p* < 0.05), greater abdominal endurance, and better Ruffier–Dickson recovery indices (6.19 ± 1.2) compared with first-division (8.07 ± 1.4) and regional players (8.97 ± 1.8). First-division athletes maintained faster 30 m sprint times (*p* = 0.004, η²*p* = 0.10), while coordination sprint performance was higher in D1–D2 players than in regional ones (*p* < 0.05, η²*p* = 0.08–0.12). No positional differences were detected for CMJ, MAS, or sprint (all *p* > 0.15, η²*p* < 0.05), likely reflecting generalized conditioning practices and uneven subgroup sizes.

**Conclusions:**

Moroccan female footballers exhibit heterogeneous performance profiles, with marked variability in jump capacity, aerobic endurance, and recovery indices across competition levels and age categories. The superior performance of second-division players in selected tests may reflect differences in training emphasis, with greater focus on core and endurance conditioning. These findings underscore the importance of individualized, data-driven preparation strategies and standardized monitoring frameworks.

**Practical implications:**

Integrating standardized physical profiling into Moroccan women’s football can optimize individualized conditioning, refine talent identification, and align domestic training models with international performance standards.

## Introduction

Football has evolved rapidly over recent decades, with players now facing substantially higher physical demands and performance expectations. While technical and tactical components remain key success factors in elite football, physical readiness has become equally essential [1, 2]. This evolution is particularly relevant in women’s football, where evidence-based athletic development programs represent a crucial step toward narrowing the performance gap with the men’s game [1, 3]. As match play becomes faster and more physically intense, a stronger emphasis has emerged on tactical and technical capabilities [4, 5]. In this context, identifying and profiling players’ physical capacities is fundamental for optimizing performance, reducing injury risk, and designing individualized training programs. Accordingly, research into standardized assessment and benchmarking of women’s football performance has gained increasing importance worldwide.

There is now a widespread scientific consensus on the value of standardized physical testing in assessing athletic profiles, enabling the identification of inter-individual variability, longitudinal progression, and position-specific benchmarks [2, 6]. However, in developing football nations such as Morocco, the empirical foundation of women’s sport science remains underdeveloped [7, 8], with fewer than 2% of peer-reviewed sport-science publications in Africa addressing women’s football over the past decade [9]. This gap underscores the lack of systematic research and performance databases dedicated to Moroccan female footballers. although institutional progress has been made, such as the professionalization of the Moroccan Women’s Championship (D1) in 2021 and the appointment of high-level technical staff for national teams [10], quantified and evidence-based performance profiling remains largely absent. Therefore, establishing scientific baselines tailored to Moroccan women’s football is crucial for guiding evidence-based athletic preparation and performance management.

Women’s football in developing contexts faces unique gender-specific challenges, including reduced access to strength and conditioning expertise, limited physiological monitoring, and fewer recovery resources. These structural constraints highlight the importance of a standardized normative database that systematically compiles physical benchmarks (e.g., speed, power, endurance, coordination, and recovery indicators). Such a database, managed within regional technical centers, could guide individualized programming, longitudinal tracking, and federation-level decision-making. Its structure would allow continuous updates and cross-sectional benchmarking across seasons, age groups, and playing positions.

Building on this national context, regional analyses can provide the empirical foundation necessary to translate structural growth into measurable athletic progress.

In recent years, women’s football in Morocco has grown markedly. The Rabat–Salé–Kénitra (RSK) regional league illustrates this progress, with a sharp rise in player registrations and affiliated clubs [11]. Yet, scientific infrastructure for performance assessment has not followed the same trajectory. Most regional teams continue to rely on traditional or experience-based training methods that lack objective information about players’ physical fitness, motor skills, and susceptibility to injury. Establishing a normative performance database could thus serve as a foundational tool for optimizing training programs and structuring athletic development pathways [8, 12]. Moreover, the integration of physical profiling into tactical and positional frameworks enhances the ability to translate physiological readiness into on-field performance, supporting collective pressing, transition speed, and recovery organization.

Accumulating evidence highlights the association of specific physical traits, such as maximal aerobic speed (MAS), linear sprint capacity, neuromuscular coordination, and explosive strength, with long-term athletic success [13, 14]. These attributes can be reliably assessed using validated, field-based protocols, such as the Vameval test, Optojump vertical jump analysis, and photoelectric-timed sprint tests, which are recognized as both accessible and effective [15, 16]. Moreover, performance profiling enables coaches to identify potential asymmetries or imbalances often linked to overuse injuries, particularly in female athletes who exhibit distinct biomechanical vulnerabilities [17, 18]. Compared with men, female footballers present distinct neuromuscular patterns, including reduced hamstring-to-quadriceps ratios, lower eccentric strength, and greater anterior cruciate ligament (ACL) injury susceptibility. These differences, combined with hormonal fluctuations and variability in recovery kinetics, justify the implementation of gender-specific profiling protocols that consider menstrual-cycle phase and sex-specific physiological adaptations.

Beyond individual diagnostics, this approach also provides practical guidance for coaches and physical trainers. These considerations directly inform the objectives of the present study, which focuses on generating normative performance references for Moroccan female footballers.

Integrating these evaluation protocols within Morocco’s regional football development framework would not only facilitate early talent identification but also support the design of training programs tailored to individual physiological profiles and position-specific demands. For example, defenders generally require greater neuromuscular robustness, midfielders depend more on aerobic capacity, and forwards prioritize acceleration and high-intensity sprinting [14, 19]. In competitive contexts, these physical attributes underpin tactical behaviors such as high pressing, counter-attack transitions, and compact defensive organization, illustrating the interdependence between conditioning profiles and strategic performance models. Understanding these differences is essential to prescribing training stimuli that optimize physiological adaptation without imposing excessive strain on athletes. Recent paradigmatic approaches also advocate for joint-by-joint and systems-based training that emphasizes inter-joint dependencies rather than isolated capacity development [20].

Considering these scientific and practical perspectives, it becomes essential to establish contextualized benchmarks adapted to Moroccan women’s football.

### Objectives and hypotheses

This study aimed to establish normative physical performance profiles of Moroccan female footballers across competitive levels and age categories, using a comprehensive, field-validated test battery encompassing sprinting, jumping, endurance, coordination, and recovery indicators. It also sought to identify inter-group differences between divisions, age groups, and playing positions to provide evidence-based reference values for national performance monitoring.

It was hypothesized that: (1) players competing at higher divisions (D1 and D2) would outperform regional-league players in most physical domains due to greater training exposure and match intensity; (2) senior and U20 players would exhibit superior performance compared with younger categories (U15–U17), reflecting maturational and neuromuscular development; and (3) positional differences would remain limited, reflecting the generalized conditioning practices prevalent in Moroccan women’s football.

## Methods

### Study design

A cross-sectional design was employed in this study to describe the physical abilities of female RSK footballers. Tests for assessing sprint time (10, 20 and 30 m), vertical and horizontal jumps, agility tests, abdominal and upper-body strength tests, Ruffier–Dickson recovery test, and the Vameval aerobic test were implemented [18, 19], thereby aiding in the development of future individualized training programs.

### Participants

The sample consisted of 109 players (*n* = 109) aged 14 to 32 years, drawn from five clubs competing in the Rabat–Salé–Kénitra (RSK) regional division series. The athletes represented all three competitive levels within the regional system. Age categories were defined according to the official classification of the Moroccan Football Federation (U15, U17, U20, and senior), ensuring comparability with national developmental structures. Players were recruited using a convenience sampling strategy, in consultation with the regional technical director, which may introduce selection bias and limit generalizability. This sampling approach was adopted due to logistical and ethical access constraints within regional football structures. However, future multicentric sampling across multiple regional leagues and national academies would help improve representativeness and external validity. Each club was invited to nominate eligible players through the regional technical directorate to ensure balanced participation across competitive divisions and playing positions. Recruitment procedures sought to reflect the diversity of the regional championship in terms of playing experience and tactical roles. The sample was selected to ensure representativeness in terms of geographical distribution and level of play. All participants had played competitive football for at least one year and provided written informed consent. Ethical approval was granted by the Institutional Ethics Committee of ibn Tofail University, in accordance with the Declaration of Helsinki.

### Experimental setting

Researchers conducted all tests sessions at the Royal Institute for Training Sports Executives (IRFC) in Salé City, which provides standardized facilities including artificial football fields, indoor gymnasiums, and professional testing equipment. Testing was performed under controlled environmental conditions: temperature maintained between 21 and 24 °C, relative humidity between 45 and 55%, on a synthetic turf surface, and in the absence of wind. To minimize circadian variability, all sessions were conducted in the late afternoon (16:00–18:00). These procedures ensured consistency across players and sessions, enhancing the reliability of the collected data [21].

### Test battery and tools

The test battery was designed to measure pivotal physical determinants of football performance, with established protocols applied for the profiling of the elite female athletes [21, 22]. The selection of tests was based on their demonstrated ecological validity in women’s football and their practicality within Moroccan regional settings. Short sprints (10–30 m) reflect match-related accelerations and transitions; jump and coordination assessments capture neuromuscular explosiveness and agility critical for duels and pressing; and the Vameval and Ruffier–Dickson tests provide robust field-based indicators of aerobic capacity and recovery with minimal equipment requirements. These tests have been widely used in female football research, ensuring both comparability and feasibility.

All assessments were supervised by five certified strength and conditioning specialists (Master in Sports Sciences, 5 + years’ experience) under the supervision of a senior researcher. Players completed all tests in a fixed order (sprints → jumps → strength → coordination → endurance → recovery) within the same session (16:00–18:00 h) after a standardized warm-up. Each test was performed in small groups of four to six players to ensure standardized pacing and recovery intervals (3–5 min between maximal efforts):

Sprint tests (10 m, 20 m, 30 m) were performed using photoelectric timing gates (Brower Timing Systems, Draper, UT, USA). Players started from a standing position with the front foot placed 0.5 m behind the first gate. Two maximal trials were performed, with the best time retained for analysis. Trials were repeated if the athlete slipped or failed to start correctly [23].

Coordination speed was assessed using a 20 m slalom sprint with cones placed at 2 m intervals. Players started from a standing position, and times were recorded with photocells at the start and finish lines. Each athlete performed two attempts, and the best time was retained [24].

Upper-limb explosive strength was evaluated through a 2-kg medicine ball throw. Athletes sat upright against a wall to prevent trunk momentum and performed two maximal throws forward. The best distance was used for analysis [21].

Lower-limb power was measured with vertical and horizontal jumps. The vertical jump was assessed using the Sargent protocol, while horizontal jump distance was measured as the best of two standing long jumps. In both cases, arm swing was permitted, and the best attempt was retained [25].

The Optojump system (Microgate, Bolzano, Italy) was used for a series of standardized jump protocols: squat jump (SJ), countermovement jumps with arms (CMJmax), countermovement jumps without arms (CMJ), drop jump (DJ), and a 15 s repeated jump test. Each athlete completed three valid trials per condition, with the best or mean performance considered depending on the test. Trials were repeated if take-off technique was invalid [26].

Trunk muscular endurance was measured using the 30-second abdominal test. Players performed as many correct sit-ups as possible within 30 s, with repetitions recorded by two trained evaluators. Trials were repeated if form was not respected [27]. Manual measures such as abdominal repetitions and heart rate counts for the Ruffier–Dickson test were independently recorded by two trained assessors and cross-checked to ensure inter-rater reliability.

Cardiorespiratory recovery was assessed using the Ruffier–Dickson recovery test, consisting of 30 squats in 45 s followed by heart rate measurements at rest, immediately post-exercise, and after 1 min of recovery. The Ruffier–Dickson index was calculated using the standard formula [28].

Aerobic endurance was evaluated through the Vameval test, performed on a synthetic pitch with 20 m shuttle runs at increasing speed (0.5 km·h⁻¹ every minute). Players ran until voluntary exhaustion, with MAS (maximal aerobic speed) determined as the last completed stage. This test is widely validated for female football populations [29, 30].

A detailed overview of the physical performance tests and their measurement protocols is presented in Table 1 below.

**Table 1:** Description of physical performance tests and measurement protocols

Test	Measured variable	Equipment	Protocol reference
10–30 m Sprint	Linear speed	Witty Gate photocell system	Haugen et al., 2019
CMJ/CMJmax/DJ/SJ/15 s	Explosive strength, reactive ability	Optojump Next System (Microgate SRL, Italy)	Bosco et al., 1983; Glatthorn et al., 2011
Sargent jump test	Explosive vertical power	Wall, chalk, measuring tape	Sargent, 1921; Requena et al., 2012
Standing long jump	Horizontal power and coordination	Measuring tape, marked take-off line	Haugen et al., 2020; Jovanovic et al., 2011
Medicine-ball throw	Upper-body power	2 kg medicine ball	Suárez-Arrones et al., 2020
Abdominal (core) test	Core endurance	Stopwatch	Nesser et al., 2008
20 m Coordination speed test	Motor coordination, agility	Witty Gate + cone grid	Reilly & Holmes, 1983; Reilly & Williams, 2003
Vameval	Maximal aerobic speed (MAS)	Audio pacing + cone-marked track	Cazorla & Léger, 1993; García-Retortillo et al., 2019
Ruffier–Dickson test	Cardiorespiratory recovery capacity	Stopwatch, heart rate monitor	Souissi et al., 2021; Abderrahman et al., 2022; Nourry et al., 2020

*CMJ* Countermovement Jump, *DJ* Drop Jump, *SJ* Squat Jump, *MAS* Maximal Aerobic Speed

Every athlete performed tests in a previously determined order, which allowed for minimal interference from fatigue, with the Vameval performed last.

All subjects completed a standardized warm-up based on the protocol of McCrary et al. [30].The routine lasted approximately 15 min and included: (i) 5 min of progressive running at low-to-moderate intensity, (ii) dynamic stretching exercises targeting major lower-limb muscle groups (quadriceps, hamstrings, gluteals, calves), (iii) mobility drills for hips and ankles, and (iv) neuromuscular activation activities such as skipping, bounding, and three short accelerations over 20 m. This sequence was designed to enhance joint mobility, increase muscle temperature, and prepare athletes for maximal efforts during testing.

Menstrual cycle phase and hormonal contraceptive use were not controlled, which represents a methodological limitation, as hormonal fluctuations can affect neuromuscular coordination, fatigue, and aerobic performance in female athletes. Future studies should integrate standardized menstrual tracking to better account for these physiological variations.

### Reliability of testing procedures

To ensure methodological rigor, test–retest reliability was assessed in a representative sub-sample of 15 players who repeated the complete testing protocol one week apart under identical conditions [31]. Inter-rater reliability for manually recorded measures (e.g., abdominal endurance test) was also verified. Reliability results (ICC and CV for each test) are reported in the Results section.

### Statistical analysis

Researchers conducted all statistical analyses using SPSS v25.0 (IBM, Armonk, NY, USA). Descriptive statistics are presented as means ± SD. Normality was assessed using the Shapiro–Wilk test and homogeneity of variance using Levene’s test. When assumptions were violated, Welch’s correction or non-parametric alternatives were applied.

Between-group comparisons were examined using one-way ANOVA and analysis of covariance (ANCOVA), with chronological age included as a covariate to account for age-related heterogeneity across competitive levels. Post hoc tests (Tukey or Games–Howell, depending on variance homogeneity) were performed to identify pairwise differences. Multiple comparisons were adjusted using the Benjamini–Hochberg false discovery rate (FDR) procedure to reduce Type I error risk.

Effect sizes were reported as partial eta-squared (η²p) for ANOVA/ANCOVA and Cohen’s *d* for pairwise comparisons, each with 95% confidence intervals. Cohen’s *d* was calculated as the mean difference divided by the pooled standard deviation and interpreted following established conventions [32, 33], where 0.2, 0.5, and 0.8 indicate small, medium, and large effects, respectively. These magnitudes were used to assess the practical significance of observed differences for training and talent identification.

Post-hoc power analyses were conducted using G*Power v3.1, confirming adequate power (≥ 0.80) for detecting medium effects (η²*p* ≈ 0.06) in age-category analyses, although reduced power was acknowledged for positional subgroup comparisons. Statistical significance was set at *p* < 0.05 (two-tailed).

## Results

The physical performance data collected from 109 female football players representing five different clubs in the Moroccan National Championship, across three levels of competition (first division, second division, and regional league), are summarized below.

### Reliability of testing procedures

Test–retest reliability was examined in a sub-sample of 15 players who repeated the complete battery under identical conditions. Intraclass correlation coefficients (ICC) and coefficients of variation (CV) confirmed high reproducibility across tests:

- Sprint tests (10, 20, 30 m): ICC = 0.91–0.95, CV = 3.8–4.5%.
- Jump tests (CMJ, CMJmax, DJ): ICC = 0.89–0.94, CV = 4.1–5.2%.
- Endurance (Vameval, MAS): ICC = 0.90, CV = 5.1%.
- Ruffier–Dickson recovery test: ICC = 0.87, CV = 5.8%.
- Abdominal endurance (30-sec test): ICC = 0.91, CV = 4.6%.
- Medicine ball throw: ICC = 0.88, CV = 5.0%.

Collectively, these values demonstrate that the testing battery provided consistent and reliable measurements suitable for performance profiling.

A detailed summary of the reliability metrics (Intraclass Correlation Coefficient – ICC, and Coefficient of Variation – CV) for each test is presented in Table 2, allowing for quick visual comparison of reproducibility across physical domains.

**Table 2:** Reliability of physical performance tests (*n* = 15)

Test	ICC (95% CI)	CV (%)	Interpretation
Sprint (10–30 m)	0.91–0.95	3.8–4.5	Excellent
CMJ/CMJmax/DJ	0.89–0.94	4.1–5.2	Excellent
Vameval (MAS)	0.90	5.1	Excellent
Ruffier–Dickson Index	0.87	5.8	Good
Abdominal Endurance	0.91	4.6	Excellent
Medicine Ball Throw	0.88	5.0	Good

Interpretation thresholds follow Koo and Li (2016): values ≥ 0.90 = excellent, 0.75–0.89 = good, 0.50–0.74 = moderate, and < 0.50 = poor reliability

*ICC* Intraclass Correlation Coefficient, *CV* Coefficient of Variation

### Descriptive and sample characteristics

Table 3 presents the age distribution, which reveals a clear stratification by competitive level, with players from the first division exhibiting the highest mean age (25.24 years), followed by those from the second division (22.68 years) and regional leagues (mean ≈ approximately 19.8 years). The age distribution across competition levels was not uniform: first- and second-division rosters were predominantly composed of senior and U20 players, whereas regional leagues were mainly composed of U15 and U17 athletes. Age-related heterogeneity was statistically accounted for by including chronological age as a covariate in ANCOVA.

**Table 3:** Descriptive statistics of age distribution among players by competitive level (*n* = 109)

Club	Mean Age (yrs)	Max Age	Min Age	SD	*N*	Level
D1	25.24	35	19	4.96	21	First Division
D2	22.68	32	17	4.09	28	Second Division
LA	20.80	27	16	2.82	20	Regional League A
LB	18.60	23	16	1.67	20	Regional League B
LC	20.05	27	14	3.53	20	Regional League C
Total/Mean	21.47	28.80	16.40	3.41	109	—

Values are presented as mean ± SD. Between-level comparisons were adjusted for age using ANCOVA. Post hoc tests (Tukey or Games–Howell) were applied according to variance homogeneity. Effect sizes are reported as partial eta-squared (η²p)

### Performance comparisons

Comparative analyses were conducted across competitive levels, age categories, and playing positions.

#### Ruffié–Dickson recovery

The Ruffier–Dickson recovery test was used to assess post-exercise cardiovascular adaptability across competitive levels (Table 4).

**Table 4:** Ruffier–Dickson recovery index values by competitive level

Competition level	*N*	HR₁ (resting)	HR₂ (post-exercise)	HR₃ (recovery)	Recovery index
First Division	21	58.7 ± 7.2	132.3 ± 12.1	89.7 ± 10.3	8.07 ± 1.4
Second Division	28	66.0 ± 8.1	114.1 ± 11.8	81.7 ± 9.2	6.19 ± 1.2
Regional League	60	71.8 ± 9.5	121.5 ± 13.4	96.4 ± 11.6	8.97 ± 1.8

Values are mean ± SD

The Ruffier–Dickson index was calculated as (HR₁ + HR₂ + HR₃ – 200)/10. Lower scores indicate better cardiovascular recovery

*HR*₁ Resting heart rate, *HR*₂ Immediate post-exercise heart rate, *HR*₃ Recovery heart rate (after 1 min)

Second-division players demonstrated the lowest Ruffier–Dickson index values (6.19 ± 1.2), followed by first-division (8.07 ± 1.4) and regional-league players (8.97 ± 1.8). On this scale, lower scores reflect better post-exercise recovery.

### Horizontal rebound

**Fig. 1:**
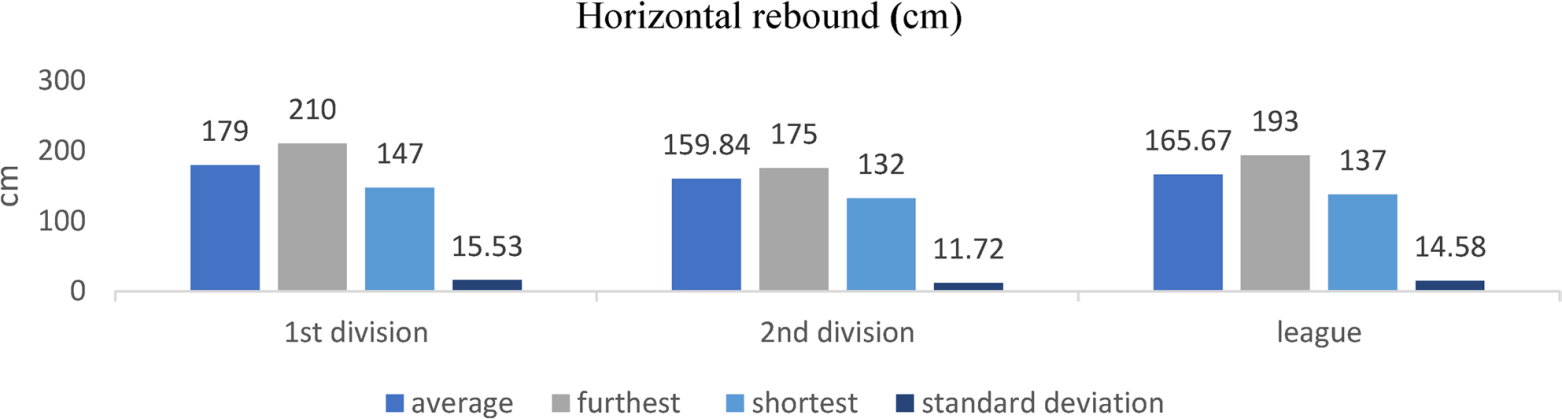
Horizontal jump performance across competitive levels (mean ± SD; cm). Sample sizes: *n* = 21 (D1), *n* = 28 (D2), *n* = 60 (Regional). Analysis: ANCOVA adjusted for age, Tukey post-hoc; *p < 0.05, FDR-adjusted

Figure 1. Second-division players demonstrated significantly greater horizontal jump distances compared to both first-division and regional players (*p* < 0.05)

#### Vertical rebound (Sargent test)

**Fig. 2:**
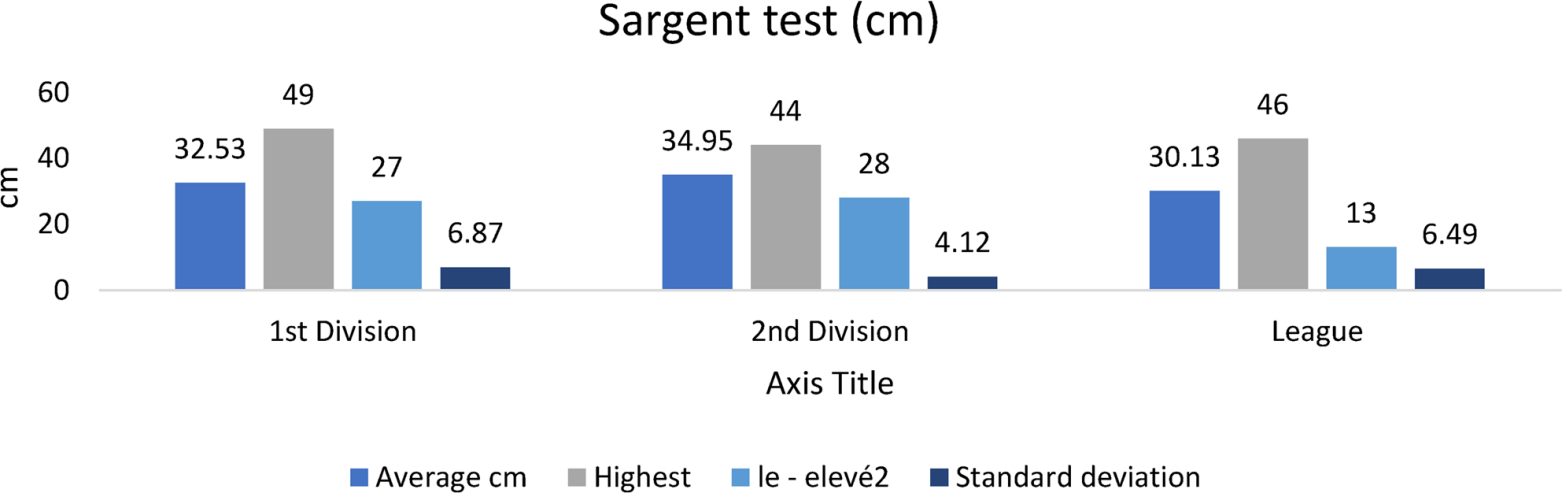
Vertical jump (Sargent test) performance across competitive levels (mean ± SD; cm). Sample sizes: *n* = 21 (D1), *n* = 28 (D2), *n* = 60 (Regional). Analysis: ANCOVA adjusted for age, Tukey post-hoc; *p < 0.05, FDR-adjusted

As illustrated in Fig. 2, second-division players achieved significantly higher vertical jump (Sargent test) values compared with both first-division and regional-league players (*p* < 0.05). Senior players also recorded greater jump heights than regional athletes (*p* < 0.05; η²*p* = 0.12), reflecting superior lower-limb explosive strength and neuromuscular efficiency at higher competitive levels.

#### Strength of the lower limbs (with optojump)

**Fig. 3:**
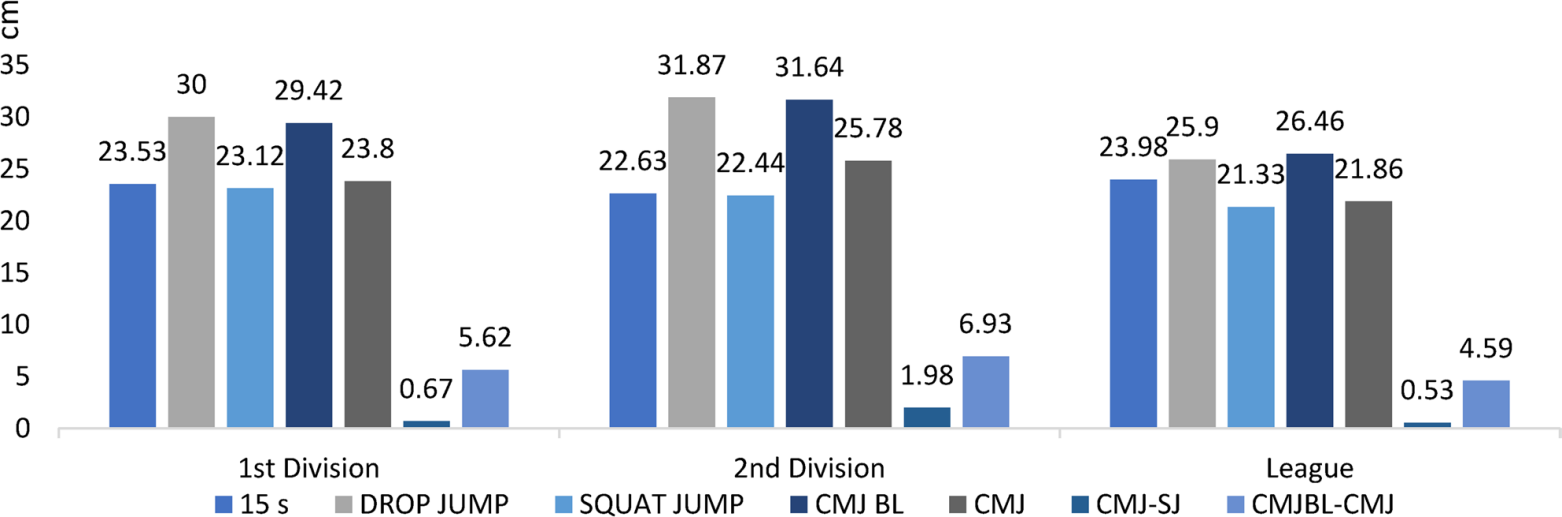
Optojump jump series (SJ, CMJ+, CMJ−, DJ, 15 s repeated) across competitive levels (mean ± SD; cm). Sample sizes: *n* = 21 (D1), *n* = 28 (D2), *n* = 60 (Regional). Analysis: ANCOVA adjusted for age, Tukey post-hoc; *p < 0.05, FDR-adjusted

Figure 3. Optojump-based assessments (SJ, CMJ+, CMJ−, DJ, and 15 s repeated jumps) revealed significant inter-level differences, with second-division players generally outperforming regional players (*p* < 0.05)

#### Abdominal strength

**Fig. 4:**
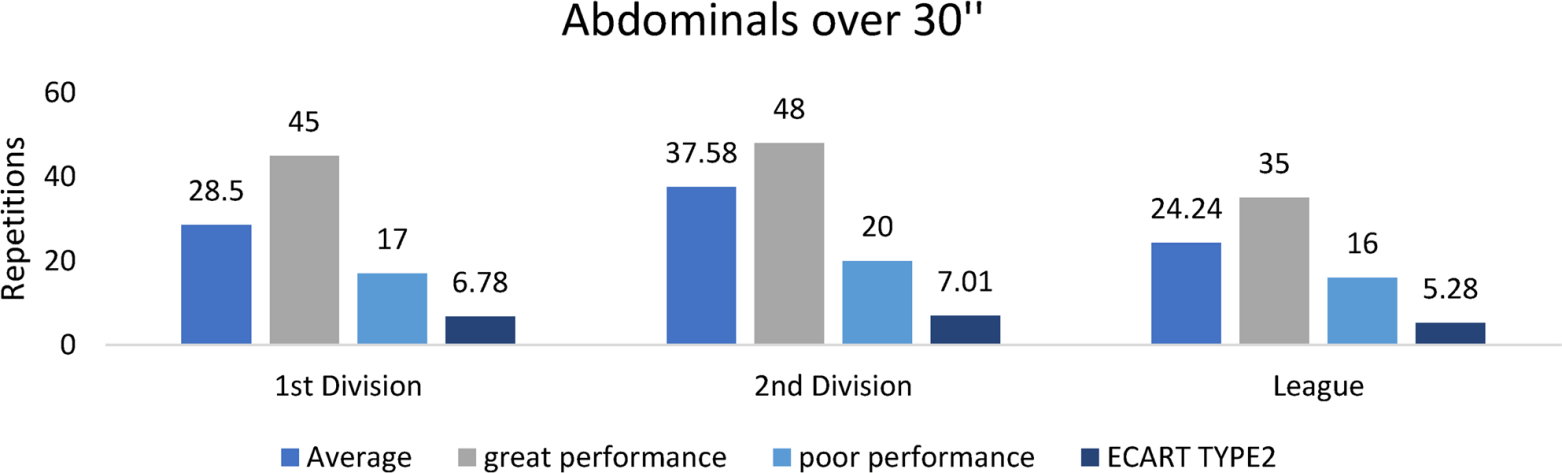
Abdominal endurance (30-second test) across competitive levels (mean ± SD; repetitions). Sample sizes: *n* = 21 (D1), *n* = 28 (D2), *n* = 60 (Regional). Analysis: ANCOVA adjusted for age, Tukey post-hoc; *p < 0.05, FDR-adjusted

Figure 4. Abdominal endurance performance, measured by the 30-second test, was significantly higher in second-division players compared with both first-division and regional players (*p* < 0.05)

### Strength of upper limbs

**Fig. 5:**
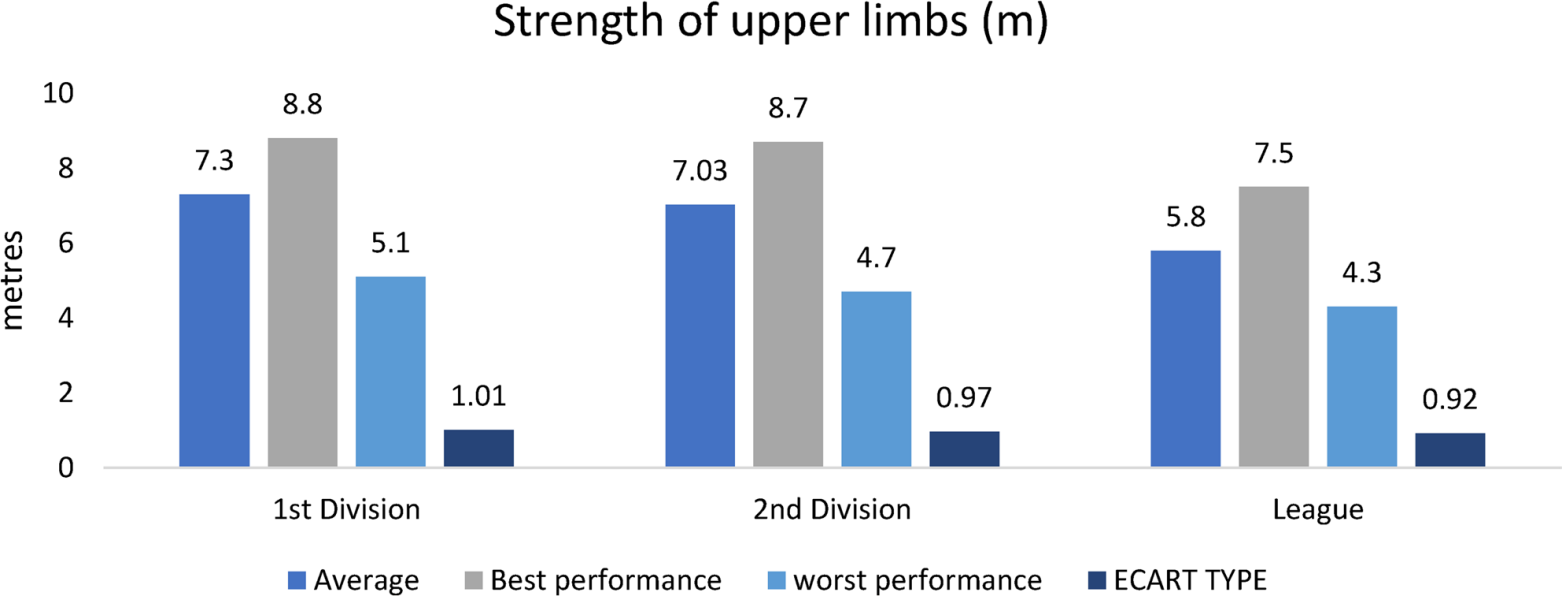
Upper-limb explosive strength (medicine ball throw, 2 kg) across competitive levels (mean ± SD; m). Sample sizes: *n* = 21 (D1), *n* = 28 (D2), *n* = 60 (Regional). Analysis: ANCOVA adjusted for age, Tukey post-hoc; *p < 0.05, FDR-adjusted

Figure 5. Upper-limb explosive strength, assessed by the 2-kg medicine ball throw, showed a progressive improvement from regional to higher divisions

Figure 5 displays the results of the upper body explosive strength test using a 2-kg medicine ball throw, a standardized measure for evaluating upper limb power in athletic contexts. The test simulates the action of a football throw-in, offering relevance to actual match situations where upper body strength supports technical actions such as long passes, aerial challenges, and defensive clearances.

### Linear velocity (three distances)

**Fig. 6:**
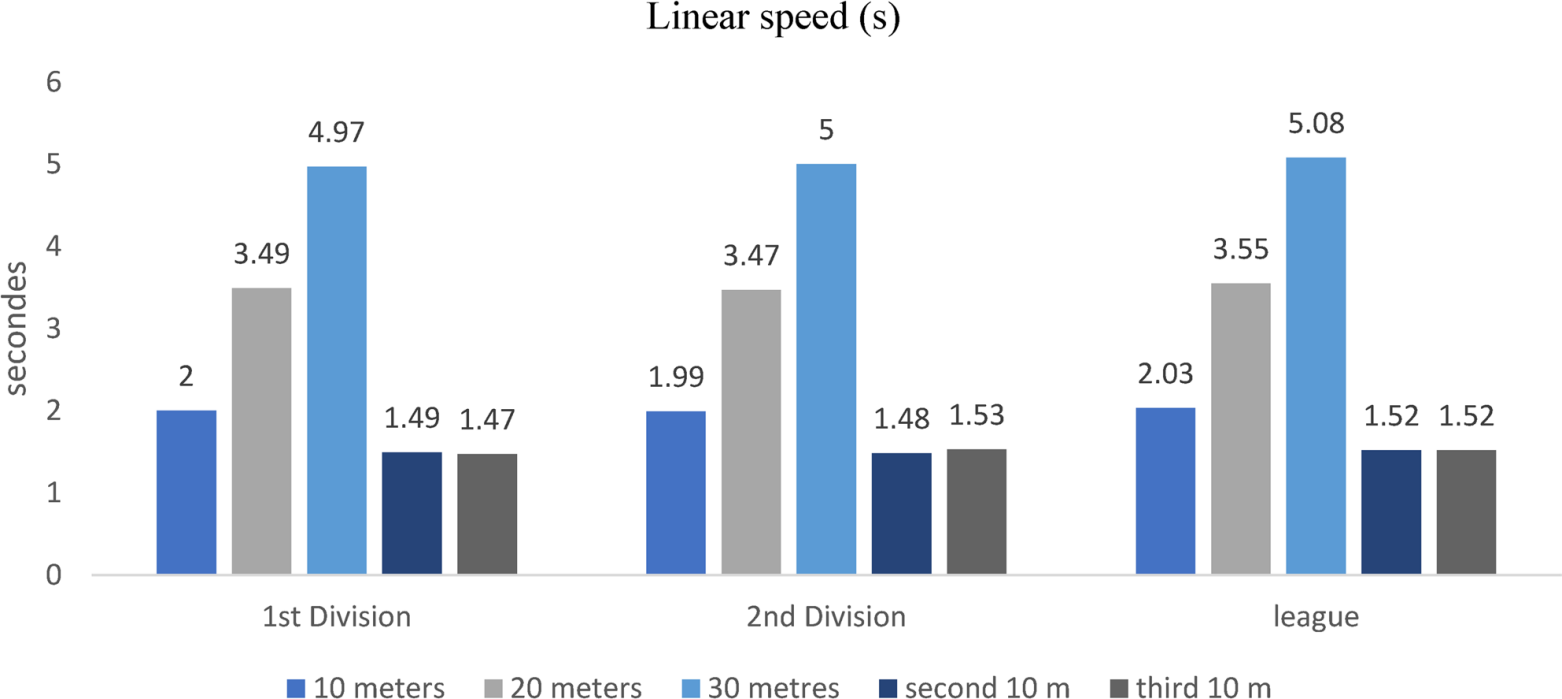
Sprint times over 10 m, 20 m, and 30 m across competitive levels (mean ± SD; s). Sample sizes: *n* = 21 (D1), *n* = 28 (D2), *n* = 60 (Regional). Analysis: ANCOVA adjusted for age; Tukey post-hoc comparisons; *p < 0.05, FDR-adjusted

Figure 6. Sprint performance revealed complementary patterns across divisions. First-division players achieved faster 30 m sprint times than other levels (*p* = 0.004, η²*p* = 0.10), while short-distance (10–20 m) acceleration did not differ significantly (*p* > 0.05).

### Speed of coordination (20 m)

**Fig. 7:**
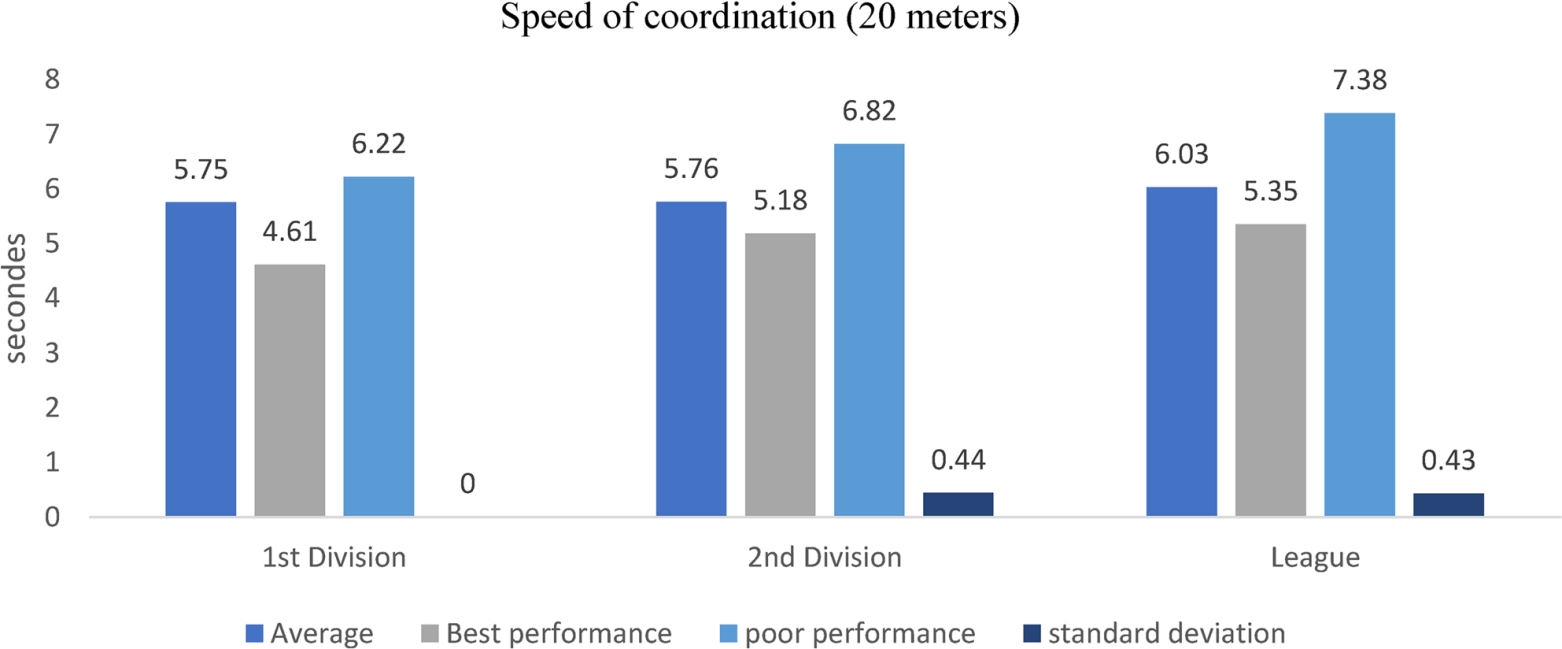
Coordination sprint (20 m slalom) across competitive levels (mean ± SD; s). Sample sizes: *n* = 21 (D1), *n* = 28 (D2), *n* = 60 (Regional). Analysis: ANCOVA adjusted for age; Tukey post-hoc comparisons; *p < 0.05, FDR-adjusted

Figure 7. Coordination sprint performance (20 m slalom) differed significantly across divisions, with D1 and D2 players outperforming regional-league athletes (*p* < 0.05, η²p values ranging 0.08–0.12), reflecting superior agility and neuromuscular control in higher-tier players.

Figure 7 illustrates the coordination sprint results for players across different competitive levels over a 20-meter slalom course. This test assesses dynamic balance, agility, and neuromuscular coordination under time pressure, critical abilities in football-specific contexts such as dribbling, pressing, and defensive recovery.

### Endurance quality and MAS

**Fig. 8:**
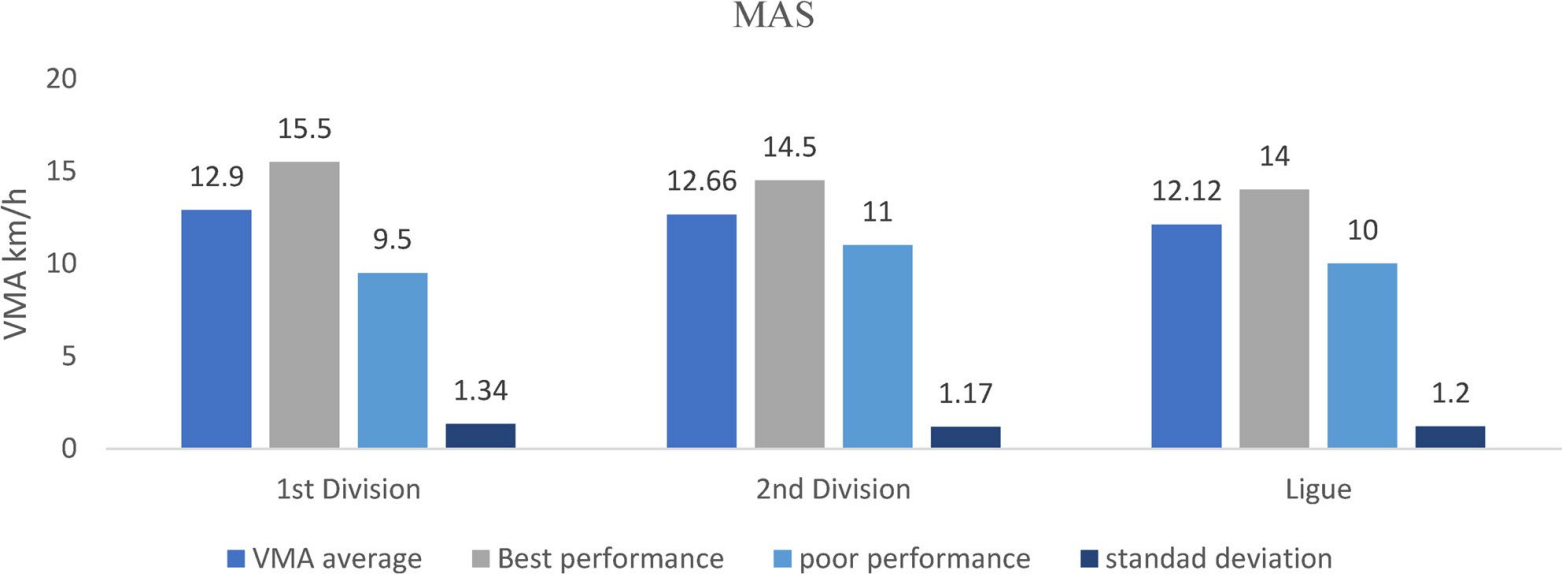
Maximal aerobic speed (MAS, Vameval test) across competitive levels (mean ± SD; km/h). Sample sizes: *n* = 21 (D1), *n* = 28 (D2), *n* = 60 (Regional). Analysis: ANCOVA adjusted for age; Tukey post-hoc comparisons; *p < 0.05, FDR-adjusted

Figure 8. Maximal aerobic speed (MAS, Vameval test) increased progressively with competitive level, with D1 and D2 players outperforming regional-league athletes (*p* < 0.05, η²*p* = 0.14), reflecting superior aerobic conditioning in higher-tier players.

### Effect size visualization

**Fig. 9:**
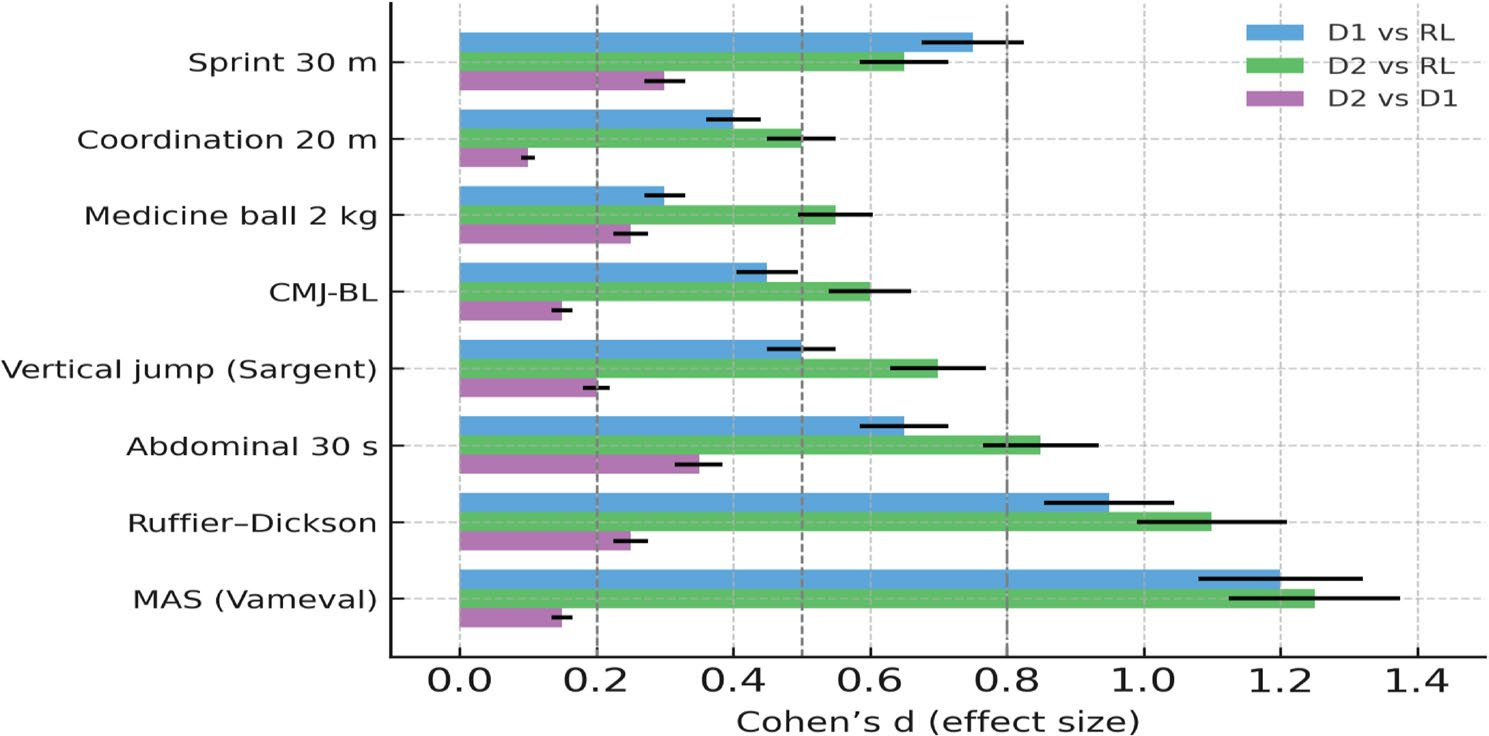
Cohen’s d effect sizes (mean ± 95% CI) illustrating the magnitude of inter-level differences in key physical performance variables among Moroccan women footballers. Thresholds for small (d = 0.2), medium (d = 0.5), and large (d = 0.8) effects are indicated by dashed vertical lines. Positive values denote superior performance of the first group in each comparison (D1 vs. RL, D2 vs. RL, D2 vs. D1). Effect sizes were computed from ANCOVA-adjusted means (SPSS v25.0)

To complement the inferential analyses, Cohen’s d effect sizes were computed to quantify the magnitude of inter-level differences across the main physical performance variables. The bar chart in Fig. 9 (mean ± 95% CI) illustrates that the largest effects emerged for aerobic capacity (MAS) and the recovery index, while moderate-to-large effects characterized lower-limb power and abdominal endurance. These patterns indicate that between-group differences are not only statistically significant but also practically meaningful for individualized conditioning, training prescription, and talent identification in Moroccan women’s football.

To provide a clearer interpretation of the practical relevance of these findings, Fig. 9 presents Cohen’s d effect sizes (mean ± 95% CI) for inter-level comparisons across sprint, jump, endurance, and recovery variables.

### Additional comparisons

To gain a more granular understanding of physical performance variation across developmental stages and tactical roles, we conducted cross-analyses based on age categories (U15, U17, U20, and seniors) and playing positions (goalkeepers, defenders, midfielders, forwards). These comparisons aimed to explore how age and field role influence specific physical capacities deemed essential in modern football performance.

### Comparison by age category

A one-way ANCOVA was performed to determine age-related differences in three key performance variables (CMJ-BL, MAS, and 10 m sprint). The results are presented in Table 5.

**Table 5:** Performance variables by age category

Variable	U15 (*n* = 20)	U17 (*n* = 25)	U20 (*n* = 30)	Senior (*n* = 34)	F(df)	*P*	η²*p*
CMJ-BL (cm)	23.4 ± 4.5	25.1 ± 4.7	27.8 ± 5.2	29.2 ± 5.8	F(3,105) = 4.03	0.021	0.105
MAS (km·h⁻¹)	10.1 ± 0.9	10.6 ± 0.8	11.2 ± 0.9	11.7 ± 0.7	F(3,105) = 7.44	0.001	0.175
10 m sprint (s)	2.07 ± 0.15	2.04 ± 0.14	2.02 ± 0.13	2.00 ± 0.14	F(3,105) = 1.96	0.147	0.053

Values are mean ± SD. Between-group comparisons tested with ANCOVA (age as covariate where applicable)

Significant age effects were found for CMJ-BL (*p* = 0.021, η²*p* = 0.105) and MAS (*p* = 0.001, η²*p* = 0.175), with senior players outperforming younger categories for both jump and aerobic performance. No significant differences were observed in 10-m sprint time (*p* = 0.147).

### Comparison by playing position

The comparative results by playing position are presented in Table 6.

**Table 6:** Performance variables by playing position

Variable	Goalkeepers (*n* = 10)	Defenders (*n* = 32)	Midfielders (*n* = 35)	Forwards (*n* = 32)	F(df)	*P*	η²*p*
CMJ-BL (cm)	25.8 ± 5.3	26.7 ± 5.1	27.1 ± 5.2	26.4 ± 5.5	F(3,105) = 0.60	0.614	0.017
MAS (km·h⁻¹)	10.7 ± 0.8	11.0 ± 0.9	11.2 ± 0.8	11.1 ± 0.9	F(3,105) = 1.77	0.157	0.048
10 m sprint (s)	2.05 ± 0.13	2.03 ± 0.14	2.02 ± 0.14	2.01 ± 0.13	F(3,105) = 0.50	0.685	0.014

Values are mean ± SD. Between-group comparisons tested with one-way ANOVA

Between-group comparisons by playing position (goalkeepers, defenders, midfielders, forwards) showed no significant differences for CMJ-BL (*p* = 0.614), MAS (*p* = 0.157), or 10-m sprint (*p* = 0.685). Effect sizes were small (η²*p* < 0.05), indicating minimal positional variation in these variables. All analyses were conducted using SPSS v25 (IBM Corp., USA). Statistical significance was set at *p* < 0.05. Effect sizes (η²p, Cohen’s d) and post-hoc power (1 − β) were reported where applicable.

## Discussion

This study established normative physical performance profiles of 109 Moroccan female footballers across three competitive levels. The results confirmed excellent test–retest reliability (ICC ≥ 0.87) and revealed that second-division players generally outperformed regional-league counterparts in jump, endurance, and recovery indices, whereas first-division athletes maintained superior sprint and high-speed profiles. These findings contribute novel reference values for female football performance within the Moroccan context. These results complement recent syntheses that consolidate benchmarks, feasibility and functional screening frameworks in women’s football [3, 14, 18]. These outcomes collectively underline how both developmental stage and competition exposure strongly influence performance outcomes, aligning with recent findings in adolescent team sports [34].

A key methodological strength of this study lies in the combination of a multi-club sample spanning three competitive levels with a reliable, field-validated test battery administered under standardized conditions, which enhances the robustness and applied relevance of the normative values generated.

### Sprint performance

Sprint ability is a critical determinant of success in women’s football, where decisive match actions such as pressing, transitions, and goal opportunities often rely on explosive acceleration and repeated sprint capacity [21, 23, 35]. In the present study, sprint performance revealed contrasting trends depending on the distance assessed. First-division players displayed significantly better 30 m sprint times (η²*p* = 0.10, *p* = 0.004), reflecting superior velocity maintenance, while second-division players achieved better results over 10–20 m accelerations, although not at a statistically significant level. No age-related differences were observed in 10 m sprint times (*p* = 0.147), suggesting that early acceleration capacity may plateau during youth development. Furthermore, no significant positional differences emerged (all *p* >0.15; η²*p* < 0.05), indicating that sprint capacity in this cohort was influenced primarily by generalized training rather than position-specific conditioning.

This lack of positional differentiation is consistent with findings from emerging football systems, where training schedules remain heavily standardized and rarely incorporate differentiated load structures across tactical roles. In such contexts, the same conditioning drills are often applied across the entire squad, regardless of positional demands, which limits the development of specific energy system adaptations or movement patterns. Consequently, the absence of positional variance in sprint performance among our cohort likely reflects a developmental stage in which physical preparation still prioritizes general fitness acquisition over role-specific performance optimization. Establishing distinct conditioning modules aligned with positional metabolic and biomechanical profiles could therefore enhance the efficiency of player development and help bridge the gap between Moroccan and elite international standards.

Although positional specialization did not emerge statistically, this likely reflects the generalized nature of conditioning within regional and national leagues, where limited resources and coaching staff constrain differentiated training loads. Nonetheless, coaches can adapt sprint conditioning frameworks by tailoring drill content and intensity to positional demands—for instance, repeated sprint and change-of-direction exercises for forwards, acceleration–deceleration transitions for defenders, and aerobic–anaerobic interval work for midfielders. Even within collective training sessions, such individualized micro-adjustments can foster more efficient role-specific adaptations and enhance tactical responsiveness during match play.

Taken together, these findings highlight the importance of integrating position-oriented sprint conditioning into Moroccan women’s football to optimize both short-distance explosiveness and velocity maintenance across competitive levels.

Applied positional conditioning: Even within generalized microcycles, coaches can embed role-specific sprint demands: (i) forwards, repeated 10–20 m accelerations with short recoveries and finish under fatigue; (ii) defenders, acceleration/deceleration waves (5–15 m) with backward/side steps and reactive starts; (iii) full-backs/wingers, longer build-up sprints (20–35 m) with cut-in COD; (iv) central midfielders, intermittent aerobic–anaerobic shuttles (10–30 m) mixing tempo runs and brief surges. Such micro-adjustments preserve collective structure while eliciting position-relevant neuromuscular and metabolic adaptations.

These trends partly mirror observations from prior studies showing that sprint ability in elite female players is more sensitive to training exposure than to chronological age [24, 33, 34]. Previous investigations highlighted the predictive value of 10–20 m sprint metrics for positional effectiveness in professional female players, whereas Mäkiniemi et al. [36] reported that forwards typically outperform defenders and midfielders in high-intensity sprinting. The absence of such positional differentiation in our sample may be attributed to the relatively small positional subgroups (e.g., goalkeepers, *n* = 10) and the lack of specialized conditioning infrastructure [37]. Moreover, the superior velocity maintenance observed in first-division players likely reflects accumulated experience, tactical demands, and greater exposure to structured sprint training, whereas the relatively younger second-division players may benefit from higher neuromuscular adaptability, enabling them to excel in shorter acceleration phases. The unexpected trend whereby second-division players performed better over short accelerations can be partly explained by load-management and fatigue accumulation at the top level, and by unequal access to conditioning resources across tiers [6, 7, 9]. Taken together, these findings highlight the importance of integrating position-oriented sprint conditioning into Moroccan women’s football to optimize both short-distance explosiveness and velocity maintenance across competitive levels.

### Jump performance

Explosive jump capacity is a key indicator of neuromuscular performance in football, underpinning repeated high-intensity actions such as aerial duels, directional changes, and pressing efforts. In the present study, vertical jump ability (CMJ-BL) was significantly higher in senior players compared with U15–U17 categories (*p* = 0.021, η²*p* = 0.105), reflecting age-related improvements in concentric power and neuromuscular coordination. Moreover, second-division players outperformed both first-division and regional-league athletes in countermovement jump height, while horizontal jump performance also followed this trend.

Optojump-derived indices indicated moderate-to-low inter-limb coordination across all levels, suggesting limitations in stretch–shortening cycle (SSC) efficiency. Such neuromuscular inefficiency may impair mechanical energy transfer between limbs, increasing the energetic cost of movement and limiting explosive strength expression during match actions such as sprint take-offs, rapid decelerations, and aerial duels. Over time, these coordination deficits can lead to asymmetrical loading patterns, reduced movement economy, and higher susceptibility to overuse injuries, particularly in female players, who tend to exhibit greater joint laxity and distinct neuromuscular activation profiles. These observations emphasize the need for coordination-oriented conditioning strategies, including unilateral plyometric drills, dynamic stabilization, and proprioceptive balance exercises, systematically integrated into weekly microcycles to enhance SSC efficiency and reduce asymmetry-related injury risk. In match play, higher inter-limb coordination supports smoother kinetic chain transfer during rapid transitions, jump–land sequences, and turning maneuvers, key components of high-intensity sequences such as pressing and counter-attacks. Over the long term, improved neuromuscular efficiency enhances both mechanical consistency and fatigue resilience, reducing the cumulative load on stabilizing joints.

These findings align with previous research showing that vertical and horizontal jumps reliably reflect lower-limb explosive power, fatigue sensitivity, and age-related neuromuscular maturation [32, 38, 39]. Slimani et al. [40] and Alba-Jiménez et al. [39] emphasized that plyometric training combined with change-of-direction drills can significantly improve reactive strength, while Cherni et al. [41] further demonstrated that loaded plyometric protocols elicit greater neuromuscular adaptations in elite female athletes.

Importantly, the moderate-to-low inter-limb coordination detected here corroborates findings from Gheller et al. [42] and Frayne et al. [43], who demonstrated that arm–leg synergy and joint coordination critically affect vertical jump efficiency. These results reinforce the need to integrate structured jump profiling and inter-limb coordination assessments into women’s football performance monitoring [38–41]. The inter-level variability observed in jump outcomes supports substantial heterogeneity in neuromuscular attributes and underscores the need for individualized and position-specific conditioning [3, 12]. The counterintuitive superiority of second-division players may be explained by differences in training emphasis and age profiles. D2 athletes were generally younger and may benefit from greater neuromuscular adaptability, while their clubs may prioritize foundational conditioning programs compared to first-division teams, where tactical demands and accumulated fatigue may reduce maximal explosive output. These patterns highlight the need to reinforce plyometric and coordination-focused training in Moroccan women’s football, not only to improve raw power but also to address asymmetries and optimize biomechanical efficiency. Regular integration of structured jump profiling could serve not only as a monitoring tool but also as a preventive framework to detect asymmetries early and optimize neuromuscular readiness throughout the season.

### Core and upper-body performance

Core stability is fundamental in football, as it supports lumbo-pelvic control, dynamic balance, and injury prevention during high-intensity actions such as sprinting, dribbling, and aerial duels. In this study, significant differences were observed between competitive levels: second-division players achieved higher abdominal endurance scores in the 30-second test compared with both first-division and regional-league athletes. Conversely, upper-body strength, measured through the 2-kg medicine ball throw, showed progressive improvements across divisions, although differences did not reach statistical significance. These trends point to underlying differences in training focus between competition levels.

The lack of statistical significance in upper-body performance may be attributed to the relatively low variance in upper-limb training exposure across divisions. In Moroccan women’s football, upper-body conditioning is often secondary to lower-limb strength and aerobic work, reflecting the predominant emphasis on technical and tactical drills in weekly microcycles. Consequently, even top-tier players may exhibit similar upper-body profiles to lower-tier athletes due to limited integration of specific upper-limb or throwing-strength sessions. Another contributing factor may be the relatively short lever-arm movements involved in the medicine ball throw (2 kg), which could limit its sensitivity to detect subtle differences in power output across heterogeneous groups.

From a practical standpoint, this highlights a missed opportunity for first-division teams to exploit upper-body training as a performance differentiator. While tactical demands and congested match schedules often restrict physical conditioning time at higher competitive levels, incorporating targeted resistance training (e.g., overhead throws, rotational power drills, and compound push–pull movements) could enhance upper-body explosiveness relevant to throw-ins, shielding, and aerial challenges. Structured integration of such exercises during low-load microcycles may optimize neuromuscular balance and contribute to overall performance resilience across the season.

Our findings corroborate earlier evidence linking trunk stability to performance and injury prevention [22, 44]. Prieske et al. [45] and Clark et al. [46] reported that inadequate core endurance is associated with compensatory movement patterns and greater incidence of lower-limb injuries, while Nesser et al. [26] demonstrated strong correlations between trunk endurance and functional performance in football-specific tasks. Similarly, Hewett et al. highlighted that reduced neuromuscular control of the trunk increases the risk of anterior cruciate ligament injury in female athletes [16]. Regarding upper-body performance, Stockbrugger & Haennel [47] validated the medicine ball throw as a proxy for explosive upper-limb power, while Loturco et al. [22] showed that balanced strength programs including trunk and upper body improve overall athletic efficiency. Hence, core and upper-body conditioning remain underdeveloped yet essential components in women’s football.

The unexpected superiority of second-division players in core endurance may reflect differences in training emphasis. and age-related neuromuscular adaptability, supporting the notion that developmental stage interacts with training strategies to shape physical outcomes [48, 49]. It is plausible that D2 teams devote greater attention to fundamental physical conditioning compared to D1 squads, where tactical and match demands dominate training routines. Furthermore, the younger age profile of D2 players may confer advantages in recovery and neuromuscular adaptability, explaining their superior results in recovery indices and abdominal endurance. These results highlight the necessity for Moroccan women’s football to reinforce systematic core training and to integrate upper-body conditioning, which remains underemphasized despite its relevance for football-specific actions such as throw-ins, shielding, and contact situations.

### Endurance capacity

Endurance performance, assessed via maximal aerobic speed (MAS) from the Vameval test, demonstrated a progressive improvement across competitive levels. Players in the first and second divisions recorded significantly higher MAS values compared with those from the regional league, whereas no meaningful differences emerged between playing positions (all *p* > 0.15). This suggests that aerobic capacity is influenced primarily by competition level and training exposure, rather than by specific positional demands in this cohort.

Maximal aerobic speed (MAS) is widely recognized as a robust indicator of match readiness in football, as it reflects the ability to sustain repeated high-intensity running and recover between efforts. Field-based aerobic assessments, such as the Vameval and the Yo-Yo intermittent recovery test, have consistently demonstrated strong correlations with total and high-intensity running distances during match play in female footballers [50]. The progressive increase in MAS across competitive levels aligns with evidence that field-based aerobic tests are applicable and informative in football when standardized appropriately [29, 47] and that high-intensity endurance capacity relates to talent pathways in female players [1].Recent comparative work has reinforced this link: Slimani et al. (2025) showed that internal biomarker responses following the Vameval test closely mirror those observed after competitive match play in elite female players, confirming that the Vameval test elicits similar physiological demands and is a valid predictor of competitive readiness [51].

Therefore, aerobic profiling remains a cornerstone for both tactical preparation and injury prevention.

Structured endurance programs based on individualized MAS zones could help regional players reduce disparities with higher divisions.

The superior MAS scores observed among first- and second-division players likely reflect more structured training loads, periodized conditioning programs, and greater exposure to high-intensity aerobic work compared with regional-level athletes. Conversely, lower MAS values among regional players may indicate developmental gaps in aerobic preparation linked to less systematic training structures. These findings emphasize the importance of implementing structured endurance periodization and individualized MAS-based conditioning modules to bridge performance disparities and enhance match readiness across competition levels.

### Recovery capacity

Recovery, evaluated by the Ruffier–Dickson test, revealed that second-division players achieved the most favorable scores compared with both first-division and regional-level athletes. Although this group exhibited slightly higher resting heart rates, their faster post-exercise normalization indicated more efficient parasympathetic reactivation and superior cardiovascular adaptability. These findings suggest that younger or less tactically burdened players may benefit from more rapid autonomic recovery.

Recovery monitoring constitutes a critical pillar of performance optimization and injury prevention in modern football. Efficient autonomic regulation, particularly the rapid parasympathetic reactivation observed after exertion, has been established as a reliable marker of aerobic fitness, training readiness, and resilience to cumulative fatigue. Heart rate variability (HRV) analysis has further demonstrated strong associations with aerobic capacity, agility, neuromuscular coordination, and sleep quality in female athletes [52]. Integrating such objective recovery markers offers a more comprehensive assessment of player readiness than workload or performance metrics alone.

Recent evidence also supports the Ruffier–Dickson index as a valid submaximal tool for evaluating cardiorespiratory recovery and autonomic function in both athletic and non-athletic populations [53]. Moreover, the increasing use of technology-driven monitoring systems has advanced recovery management in football. For instance, Ardigò et al. (2020) demonstrated that the SuperOp™ device exhibits strong external responsiveness in detecting post-exercise recovery dynamics, highlighting the growing shift toward data-informed and individualized recovery protocols in sports science [54, 55].

The superior recovery profiles in second-division players may be explained by several factors: (i) younger average age and therefore greater autonomic adaptability; (ii) less cumulative match congestion and tactical fatigue compared with first-division players; and (iii) potential emphasis on foundational conditioning in D2 training environments. Collectively, these observations highlight the importance of integrating recovery-focused strategies and innovative monitoring technologies into athlete management frameworks [56], such as low-intensity aerobic work, controlled breathing exercises, sleep hygiene education, and HRV monitoring, within weekly microcycles. In practical application, HRV monitoring can be implemented through daily morning assessments using validated smartphone applications or wearable sensors, providing individualized readiness scores that guide day-to-day training adjustments. A consistent decline in HRV over consecutive days may signal excessive fatigue or insufficient recovery, prompting a reduction in training load or inclusion of active recovery sessions. Similarly, integrating recovery tracking tools such as the Wellness Questionnaire (fatigue, soreness, sleep quality, stress) and short submaximal heart rate tests can offer complementary insights into each athlete’s physiological state. These measures allow coaches to tailor session intensity and volume within microcycles, aligning internal recovery markers with external load metrics (e.g., GPS-based running volume, high-speed efforts). The combination of HRV and subjective recovery monitoring can thus optimize periodization and prevent maladaptation, particularly during congested match schedules. Embedding such practices in Moroccan women’s football could enhance both training efficiency and resilience against overtraining.

The recurrent advantage observed among second-division players across various performance domains (jumping, core endurance, and recovery) appears to reflect broader structural dynamics rather than isolated test outcomes. This trend suggests that D2 athletes may currently occupy an optimal developmental window, combining youth-related physiological plasticity with structured but less congested training loads, which favors general fitness expression during standardized assessments. Conversely, first-division players operate within more tactically demanding and congested competitive environments, which can mask their maximal physical potential despite greater technical and strategic proficiency. When interpreted holistically, these results emphasize the need for context-specific conditioning frameworks that preserve physical freshness and neuromuscular adaptability across competitive tiers.

### Practical implications for Moroccan women’s football

From a practical perspective, the normative data generated in this study offer a valuable foundation for designing and monitoring evidence-based training within Moroccan women’s football. Coaches and conditioning staff can use these reference values to establish individualized benchmarks for sprinting, jumping, aerobic capacity, and recovery, enabling the creation of performance targets tailored to age, competitive level, and playing position.

Integrating standardized field-based tests, such as Optojump jump protocols, sprint timing gates, the Vameval test, and the Ruffier–Dickson index, within seasonal training cycles would allow systematic tracking of players’ development and early detection of performance stagnation or fatigue. These procedures could be incorporated into the technical and medical structures of the Royal Moroccan Football Federation (FRMF) to support data-driven talent identification and performance management [3, 12, 18].

At the club level, implementing regular assessment batteries would facilitate communication between coaches, fitness trainers, and medical staff, ensuring better alignment between training stimuli and physiological adaptations. In regional academies, the application of such normative benchmarks could guide long-term athletic development, while at the national level, a centralized digital database could enhance comparative monitoring across divisions.

Ultimately, these practices would help reduce inter-regional disparities, optimize player readiness, and promote a culture of performance analytics aligned with international standards [19, 22, 35]. By embedding evidence-based monitoring and individualized conditioning into women’s football programs, Morocco can accelerate its progression toward continental and global competitiveness.

Additionally, future work could apply multivariate similarity techniques, such as Euclidean or Mahalanobis distance-based clustering, to explore latent player archetypes and multidimensional performance patterns across competitive levels and playing positions.

A further limitation concerns the lack of comparative datasets from Muslim women athletes in North Africa, the Middle East, or other Islamic contexts. This gap reflects both the scarcity of sport-science research involving female populations in these regions and broader sociocultural barriers that still restrict women’s participation in competitive sport. By providing normative reference values for Moroccan women footballers, the present study contributes to filling this void and supports ongoing efforts to demarginalize women in sport. In practical terms, these benchmarks can serve as a foundation for cross-country collaborations between federations in Muslim-majority nations, facilitating shared performance standards and promoting greater visibility and recognition of women’s athletic achievements.

### Limitations

This study is not without limitations. First, the use of a convenience sample recruited in consultation with the regional technical director introduces potential selection bias and restricts the generalizability of findings beyond the participating clubs. Future research should consider stratified or multicentric sampling designs across regions to ensure broader representativeness of the national female football population.

Second, the cross-sectional design prevents causal inference and does not allow longitudinal monitoring of developmental trajectories. Third, the absence of menstrual cycle phase or contraceptive-use control represents a major limitation, as hormonal fluctuations are known to influence neuromuscular performance, endurance capacity, ligament laxity, and recovery dynamics in female athletes.

Fourth, the Ruffier–Dickson index was interpreted using thresholds derived from general-population studies, which may not fully capture sport- or sex-specific adaptations. Finally, the small sample sizes of some subgroups (e.g., only 10 goalkeepers) may have limited the statistical power to detect subtle positional differences.

Although the inclusion criterion of at least one year of competitive experience ensured basic familiarity with football training, it may not fully capture long-term adaptation effects. Future research should consider stricter criteria (e.g., ≥ 3 years of continuous participation) to strengthen internal validity.

### Future research directions

Future research should build on these findings through longitudinal and multicentric designs to better capture seasonal variations and training adaptations in Moroccan female footballers. Expanding samples to include multiple regional leagues and youth academies would improve representativeness and allow the development of position- and age-specific performance norms.

Integrating GPS tracking, match-load indicators, and psychophysiological measures (e.g., wellness, sleep, hormonal monitoring) could provide a more holistic understanding of how training and competition demands affect performance and recovery.

Further, studies should systematically account for menstrual-cycle phases and contraceptive use, following current methodological standards for female athlete research [57].

At the practical level, developing a centralized performance database under the Royal Moroccan Football Federation (FRMF) could facilitate national benchmarking and foster collaboration between clubs, researchers, and medical departments.

Finally, future investigations should evaluate the effectiveness of individualized conditioning interventions, such as plyometric, speed, or recovery-based programs, on longitudinal improvements in female football performance and injury prevention [12, 45, 53].

## Conclusion

This study presents one of the first comprehensive and multidimensional performance datasets for Moroccan female footballers, encompassing sprint, jump, coordination, endurance, and recovery capacities across competitive levels. It establishes reliable normative benchmarks and confirms significant variability across age and division, with senior players showing superior countermovement jump and aerobic performance, while acceleration remained relatively stable across groups.

Beyond these descriptive insights, the study highlights the complex interaction between training exposure, competition demands, and developmental context, particularly the counterintuitive advantage of second-division players in selected metrics, underscoring the need for data-driven monitoring in emerging football ecosystems.

The normative benchmarks generated here provide a valuable reference for coaches, conditioning specialists, and federation staff to design individualized training plans, monitor progress, and support talent identification. Embedding these standardized testing protocols within regional academies and national programs could enhance player development pathways and align domestic preparation models with international standards.

Overall, this study offers a scientific foundation for evidence-based training and performance management in Moroccan women’s football, contributing to its ongoing professionalization and competitiveness on the African and global stage.

## Data Availability

The datasets generated and analyzed during the current study are available from the corresponding author upon reasonable request.

## Abbreviations

Abbreviation: Definition
ANCOVA: Analysis of Covariance
ANOVA: Analysis of Variance
CMJ: Countermovement Jump
CMJ max: Countermovement Jump with Arm Swing (Maximum)
CMJ-BL: Countermovement Jump with Arm Swing (Baseline)
CV: Coefficient of Variation
D1: First Division
D2: Second Division
DJ: Drop Jump
FDR: False Discovery Rate
HR: Heart Rate
HR₁: Resting Heart Rate
HR₂: Post-exercise Heart Rate
HR₃: Recovery Heart Rate (after 1 min)
HRV: Heart Rate Variability
ICC: Intraclass Correlation Coefficient
IRFC: Royal Institute for Training Sports Executives
MAS: Maximal Aerobic Speed
RSK: Rabat–Salé–Kénitra
SD: Standard Deviation
SJ: Squat Jump
SSC: Stretch–Shortening Cycle
η²p: Partial Eta-Squared
